# Supplementary material for: Pellino1 regulates reversible ATM activation via NBS1 ubiquitination at DNA double-strand breaks
Source: Nat Commun. 2019 Apr 5;10:1577. doi: 10.1038/s41467-019-09641-9 (PMC6450972; doi:10.1038/s41467-019-09641-9)
Supplement: Supplementary file 1 — Supplementary Information [file 41467_2019_9641_MOESM1_ESM.pdf]

Supplementary Information

Pellino1 regulates reversible ATM activation via NBS1 ubiquitination at DNA double strand breaks

Ha et al.

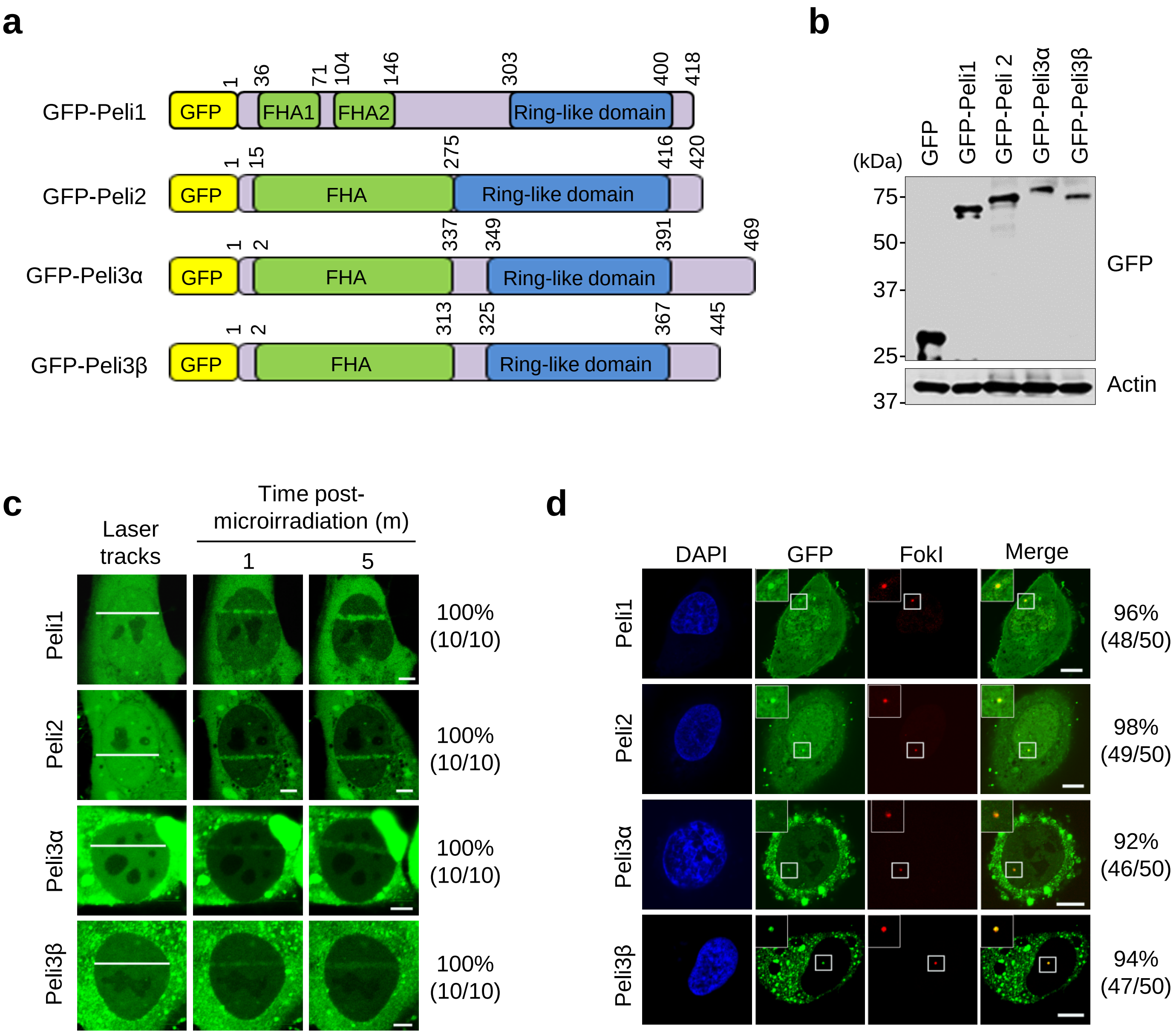

Supplementary Fig. 1 Peli family accumulates at DNA damage sites. **a, b** Diagrams of GFP-tagged Peli1, -Peli2, -Peli3α, and -Peli3β (**a**). 293T cells were transfected with plasmids encoding GFP-Peli1, GFP-Peli2, GFP-Peli3α, and GFP-Peli3β plasmid. After 48 hr, cells were harvested and cell lysates were analyzed by immunoblotting with anti-GFP and anti-actin antibodies (**b**). **c** U2OS cells were transfected with GFP-fused Peli1, Peli2, Peli3α, or Peli3β and treated with BrdU (10 μM) for 30 hr followed by laser microirradiation. Scale bar, 10 μM. **d** mCherry-LacI-FokI was co-transfected with indicated plasmids encoding GFP-Peli1, GFP-Peli2, GFP-Peli3α, or GFP-Peli3β into U2OS DSB reporter cells (U2OS 2-6-3). After 48 hr, live cell imaging was performed with confocal microscopy. Scale bar, 10 μM.

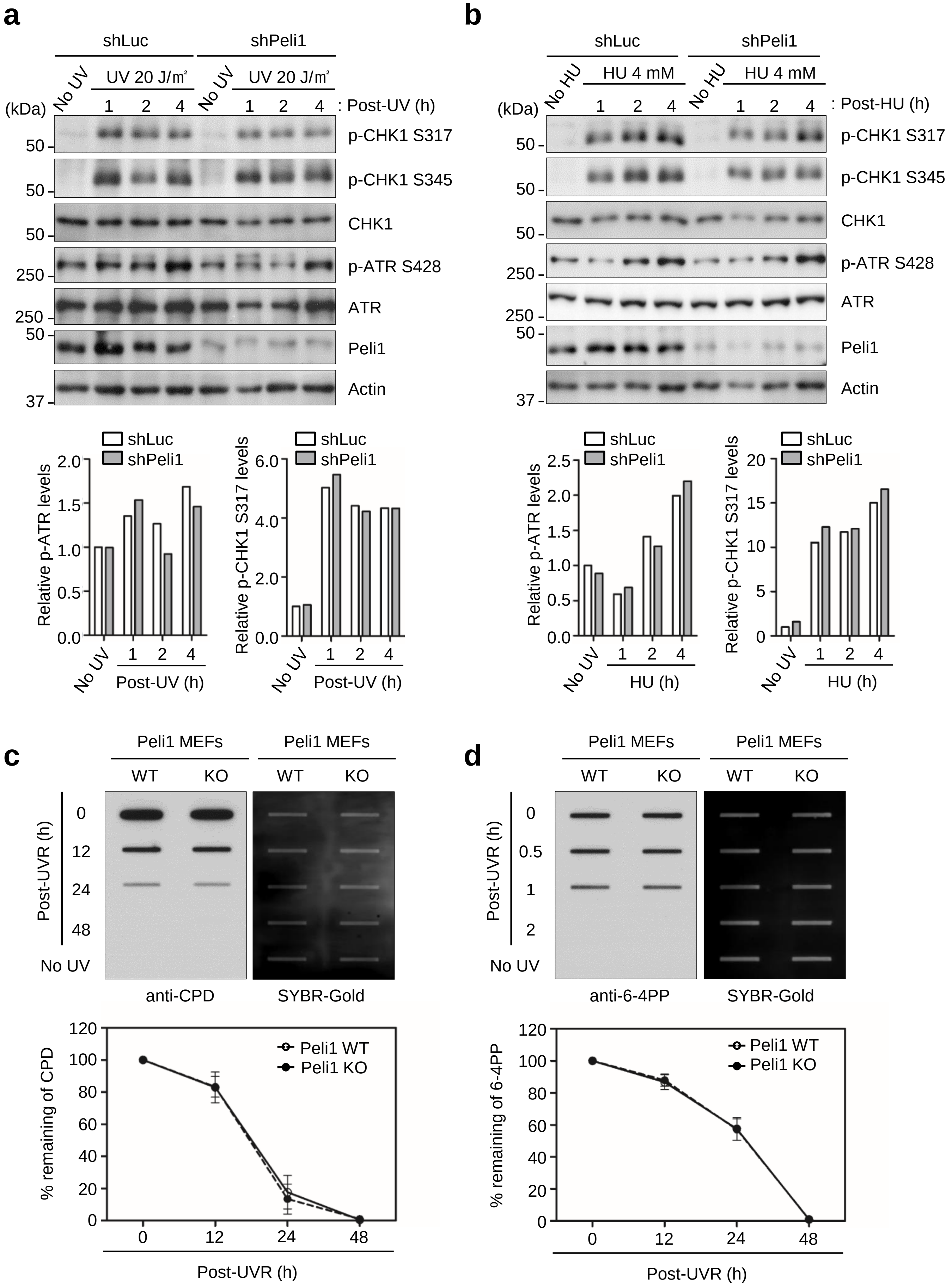
a
shLuc
shPeli1
No UV
UV 20 J/㎡
: Post-UV (h)
(kDa)
p-CHK1 S317
p-CHK1 S345
CHK1
p-ATR S428
ATR
Peli1
Actin
Relative p-ATR levels
Relative p-CHK1 S317 levels
Post-UV (h)
b
HU 4 mM
No HU
: Post-HU (h)
HU (h)
c
Peli1 MEFs
WT
KO
Post-UVR (h)
anti-CPD
SYBR-Gold
% remaining of CPD
Peli1 WT
Peli1 KO
d
anti-6-4PP
% remaining of 6-4PP

Supplementary Fig. 2 Peli1 is dispensable for ATR activation in single-strand breaks. **a, b** Peli1 is not required for ATR activation in response to UV or HU treatment. U2OS cells were transfected with shRNA for control or Peli1 knock down. At 24 hr after transfection, cells were irradiated with 20 J/m^2 of UVC (**a**) or treated with 4 mM of HU (**b**). After indicated recovery (for UVR) or treatment (for HU) time, cells were harvested and lysed for immunoblot analysis. Phosphorylation of CHK1 and ATR was assessed by immunoblotting with indicated antibodies. Graphs show relative band intensities for phospho-CHK1 and phospho-ATR normalized to CHK1 and ATR, respectively. **c, d** Downregulation of Peli1 has no effect on nucleotide excision repair activity evoked by UV irradiation. Wild type and Peli1 knockout MEF cells were exposed to 20 J/m^2 of UVC. Recovery was allowed for the indicated time. Genomic DNAs obtained at each time point were analyzed to detect residual CPD (**c**) or 6–4PP (**d**) lesions using DNA slot blot repair assay with UV damage-specific monoclonal antibodies. The membrane counterstained with SYBR-Gold was used as a loading control of genomic DNA.

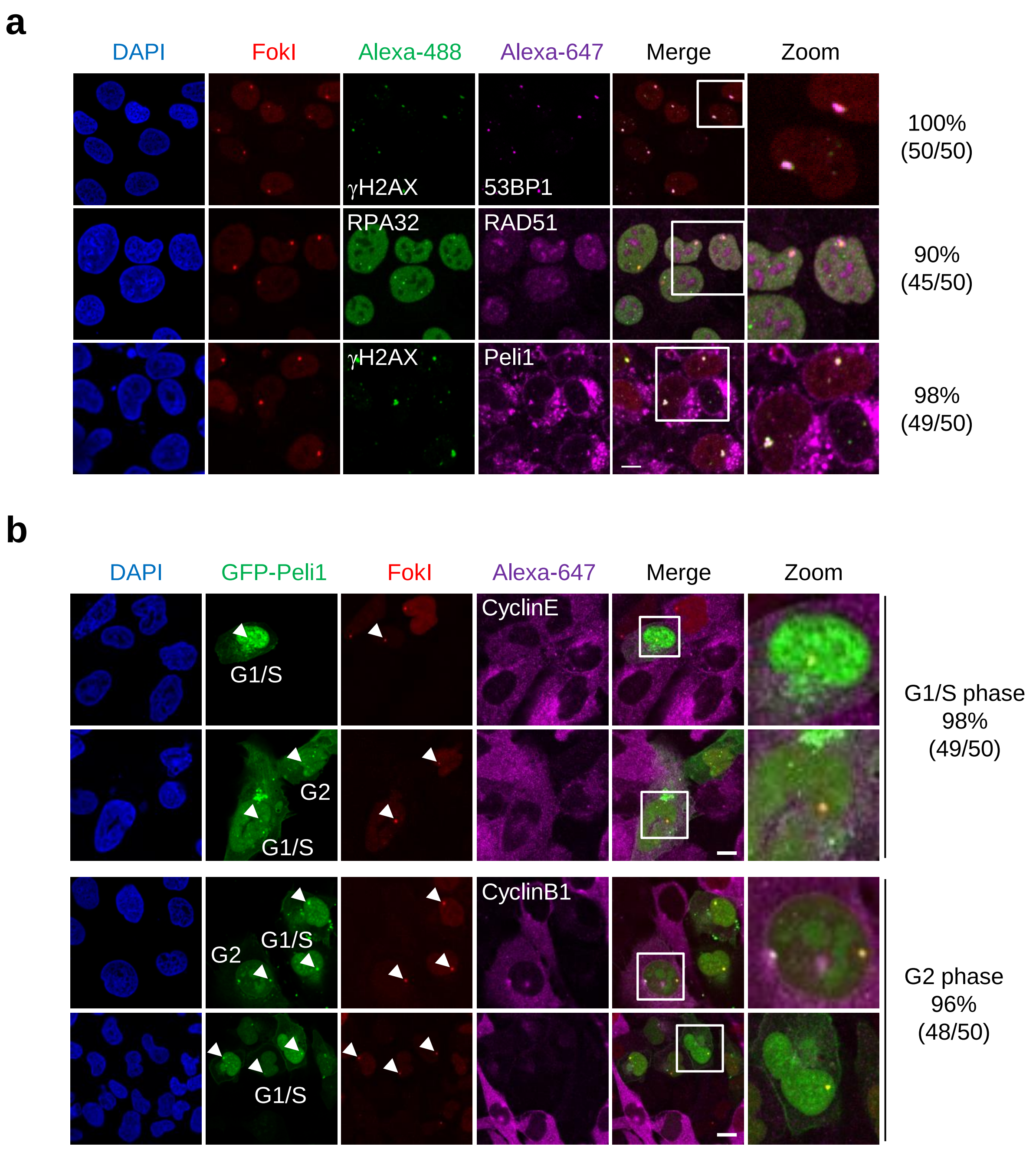

Supplementary Fig. 3 Peli1 is recruited at DNA damage sites during cell cycle progression. **a** mCherry-LacI-FokI plasmid was transfected into U2OS 2-6-3 cells. After 48 hr, cells were fixed and immunostained with indicated antibodies. Scale bar, 10 μM. **b** GFP-Peli1 and mCherry-LacI-FokI constructs were co-transfected into U2OS 2-6-3 cells. After 48 hr, cells were fixed and immunostained with cell cycle-dependent antibody, Cyclin E or Cyclin B1. Scale bar, 10 μM.

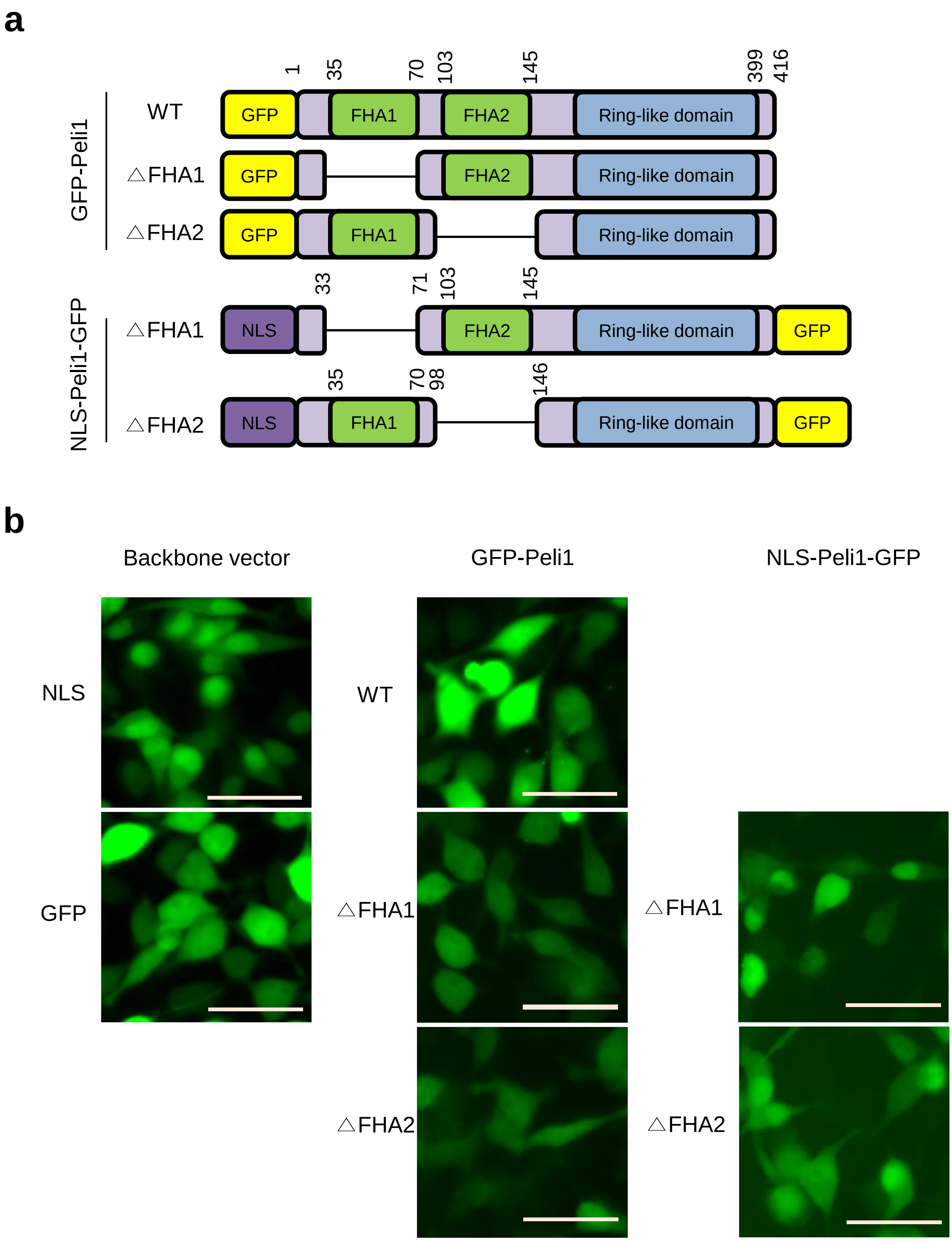

Supplementary Fig. 4 Subcellular distribution of Peli1 FHA1 deletion or FHA2 deletion by adding nuclear localization signal (NLS). **a** Schematics of various GFP-Peli1 WT, FHA1 deletion, FHA2 deletion, NLS-GFP-Peli1 WT, FHA1 deletion, and FHA2 deletion constructs. **b** Each construct encoding GFP-Peli1 or NLS-GFP-Peli1 WT or mutant was transfected into HeLa cells. At 36 hr post-transfection, cells were analyzed with an immunofluorescence microscope. Scale bar, 50 μM.

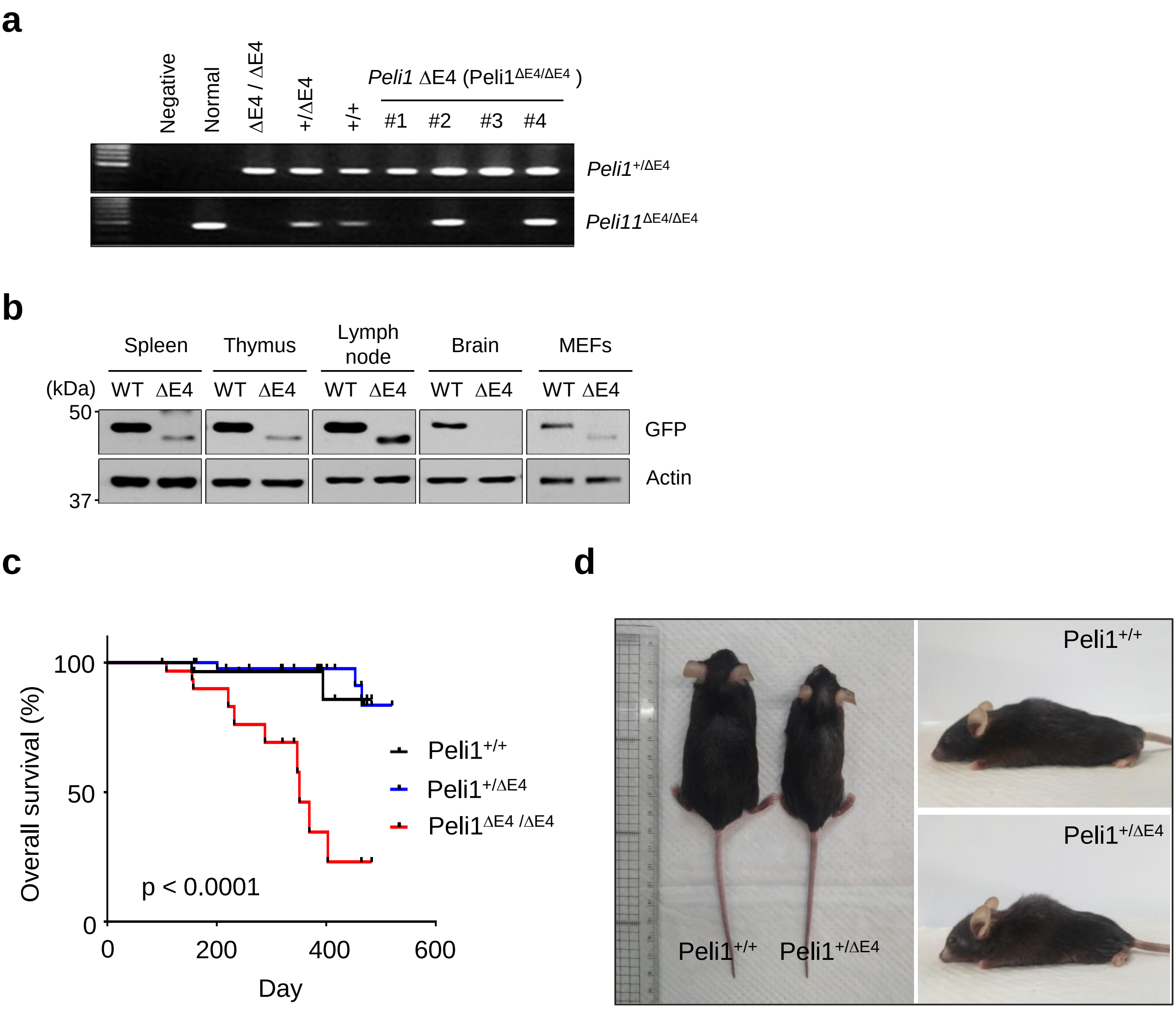

Supplementary Fig. 5 Generation of Peli1 wild type (WT) and exon 4 (E4) homozygous mouse embryonic fibroblast (MEF) cells and survival of WT, E4 heterozygous and E4 homozygous mice. **a** Genotyping PCR analysis to detect Peli1 WT, Peli1 heterozygous, and homozygous mice. **b** Immunoblotting analysis of Peli1 in various tissues and cultured primary cells of Peli1 WT and Peli1 E4 homozygous mice. Actin was used as a loading control. **c** Kaplan-Meier curves of overall survival for Peli1 WT (n=20), Peli1 E4 heterozygous (n=15) and Peli1 homozygous (n=17) mice from 3 independent founder lines. **d** Representative images of Peli1 WT and E4 heterozygous mice at 33-34 weeks of age. This study was reviewed and approved by the institutional animal care and use committee of Sungkyunkwan university school of medicine.

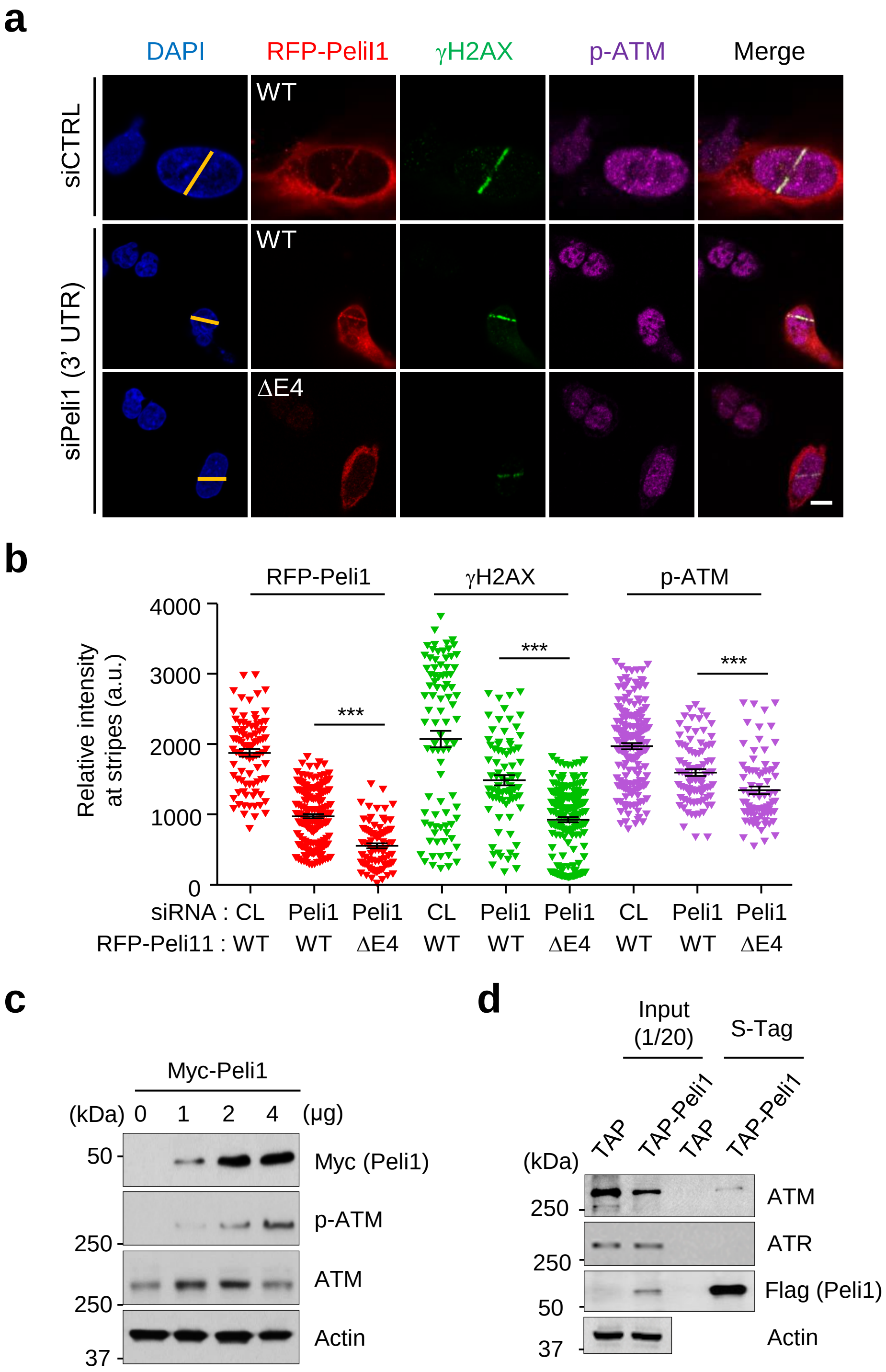

Supplementary Fig. 6 Peli1 interacts with ATM and promotes ATM activation and γH2AX signals at DSB sites. **a, b** U2OS cells were transfected with Peli1 3'-UTR-targeting siRNA or control siRNA. RFP-Peli1 wild type or E4 truncated construct was then transfected again into cells with BrdU (10 μM). At 48 hr post-transfection, RFP positive cells were microirradiated and fixed 10 min later. Laser stripes were detected with anti-γH2AX and anti-p-ATM antibodies. Scale bar, 10 μM (**a**). Quantitative analysis for localization signals of RFP-Peli1 and indicated antibodies at laser stripes. Plotted values represent mean±s.e.m. of 20 cells. Student's *t*-test was used for statistical analyses (**b**). **c** 293T cells were transfected with Myc-Peli1 plasmids (0, 1, 2, 4 μg). At 48 hr post-transfection, cell lysates were analyzed by immunoblotting with anti-Myc, anti-ATM, anti-p-ATM, and anti-actin antibodies. **d** 293T cells were transfected with TAP (control) or TAP-Peli1 (Flag-tagged Peli1). At 36 hr post transfection, cells were harvested and isolated through S-tag pull down assay. Bound proteins were immunoblotted with anti-Flag, anti-ATM, anti-ATR, and anti-actin antibodies.

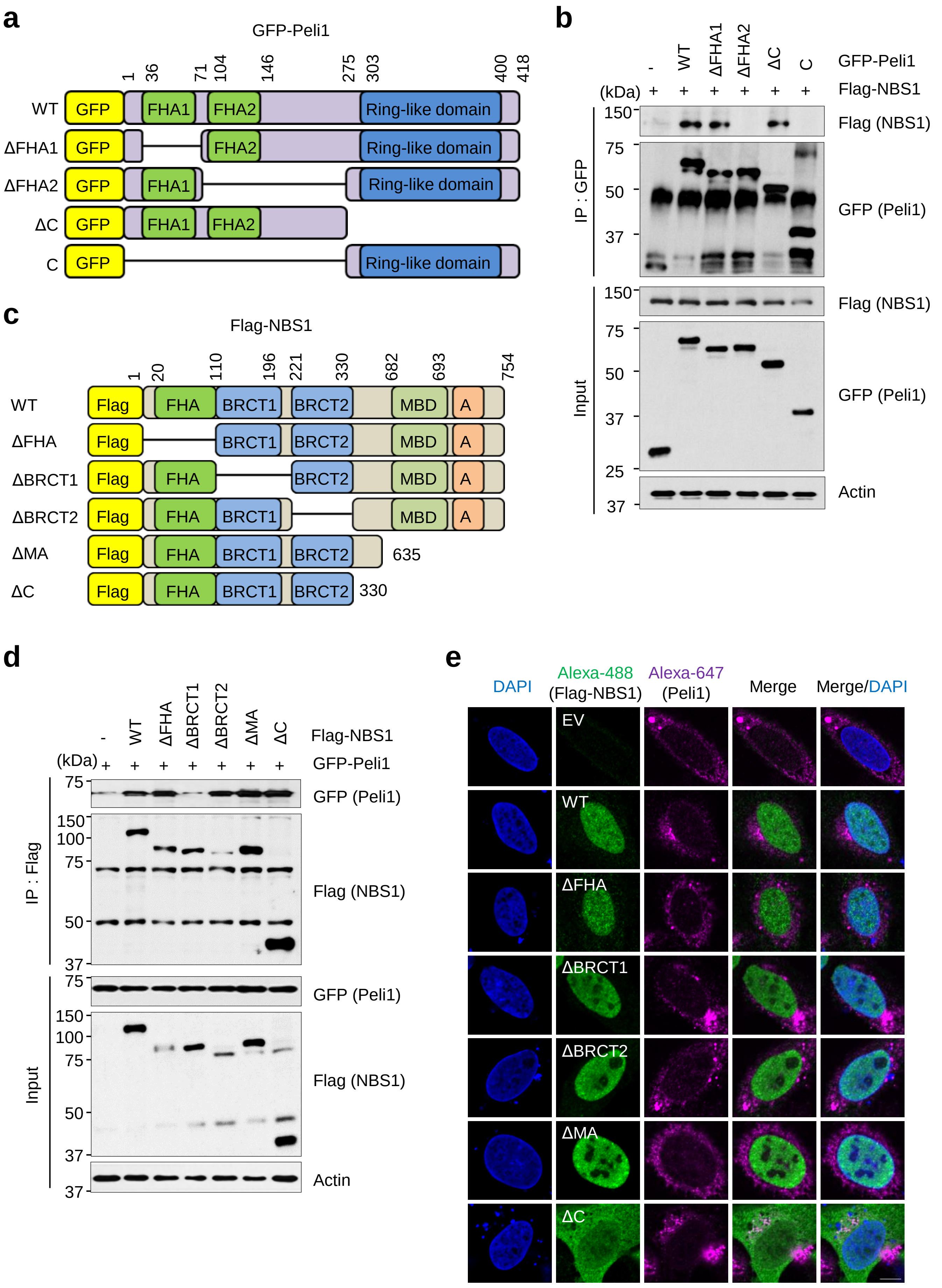

a
GFP-Peli1
WT
ΔFHA1
ΔFHA2
ΔC
C
GFP
FHA1
FHA2
Ring-like domain
1 36 71 104 146 275 303 400 418
b
GFP-Peli1
Flag-NBS1
- WT ΔFHA1 ΔFHA2 ΔC C
(kDa)
IP : GFP
Input
Flag (NBS1)
GFP (Peli1)
Actin
c
Flag-NBS1
WT
ΔFHA
ΔBRCT1
ΔBRCT2
ΔMA
ΔC
Flag
FHA
BRCT1
BRCT2
MBD
A
1 20 110 196 221 330 682 693 754
635
330
d
Flag-NBS1
GFP-Peli1
- WT ΔFHA ΔBRCT1 ΔBRCT2 ΔMA ΔC
(kDa)
IP : Flag
Input
GFP (Peli1)
Flag (NBS1)
Actin
e
DAPI
Alexa-488 (Flag-NBS1)
Alexa-647 (Peli1)
Merge
Merge/DAPI
EV
WT
ΔFHA
ΔBRCT1
ΔBRCT2
ΔMA
ΔC

Supplementary Fig. 7 Peli1 interacts with NBS1 via BRCT domain. **a** Schematics of various Peli1 truncated mutants. **b** Flag-NBS1 was co-transfected with indicated plasmids encoding GFP and GFP-Peli1 WT, FHA1 deletion, FHA2 deletion , C-terminal deletion, or C-terminal mutant. At 36 hr, cells were harvested and then immunoprecipitated with Flag antibody and subjected to immunoblot with anti-Flag, anti-GFP, and anti-actin antibodies. **c** Schematics of various NBS1 truncated mutants. **d, e** GFP-Peli1 WT was co-transfected with indicated plasmids encoding Flag and Flag-NBS1 WT, FHA deletion, BRCT1 deletion, BRCT2 deletion, MA deletion, or C-terminal deletion mutant. At 36 hr, cells were harvested and then immunoprecipitated with Flag antibody and subjected to immunoblot with anti-Flag, anti-GFP, and anti-actin antibodies (**d**). Cells were transfected with FLAG empty vector (EV), FLAG-NBS1 WT, and FLAG-NBS1 truncated mutants into U2OS cells. At 48 hr post transfection, cells were fixed and immunostained with anti-FLAG and anti-Peli1 antibodies (**e**). Scale bar, 10 μM.

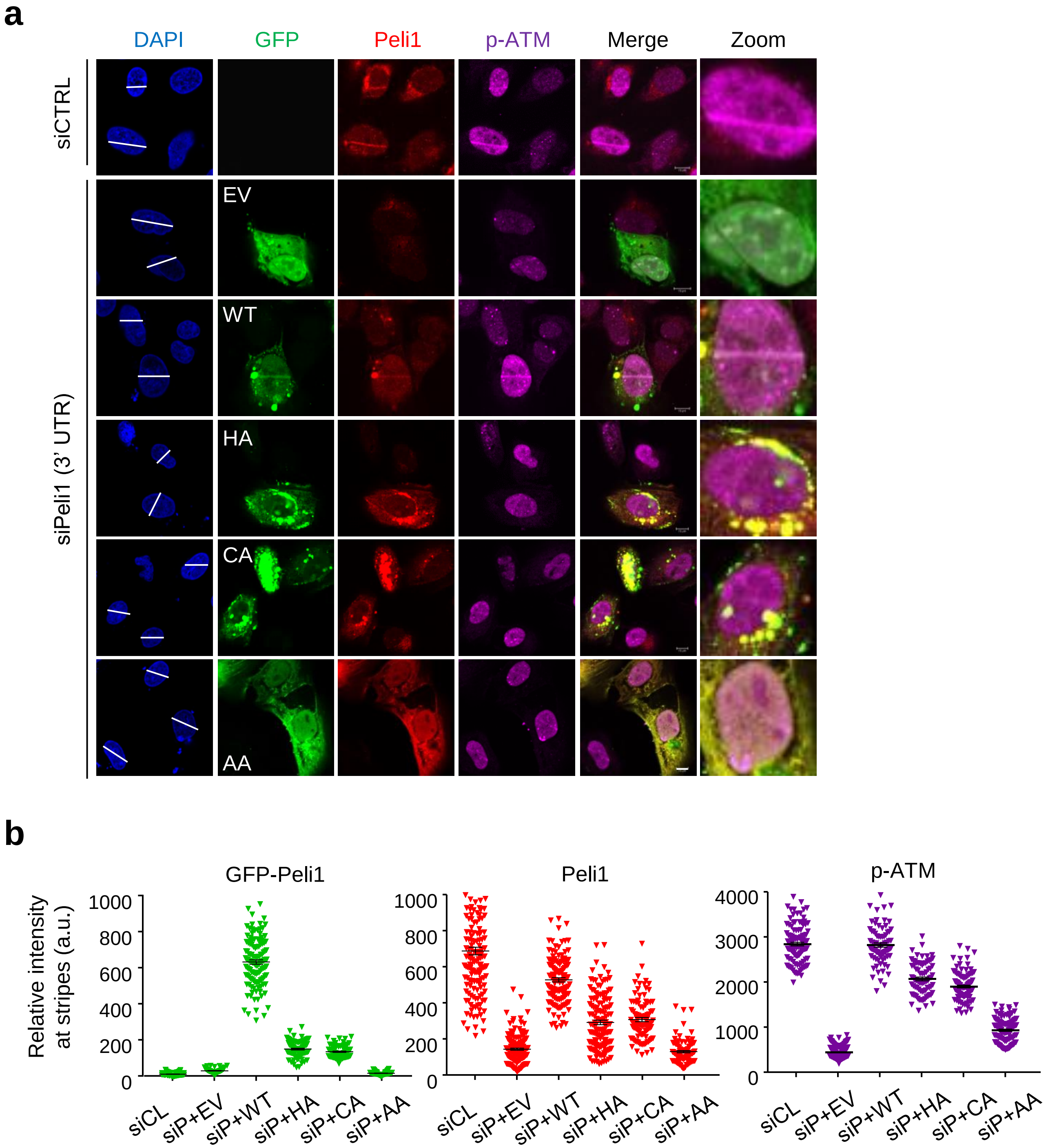

Supplementary Fig. 8 Peli1 ligase activity and phosphorylation are required for phospho-ATM accumulation at laser stripes. **a** U2OS cells were transfected with Peli1 3'-UTR-targeting siRNA or control siRNA. After 24 hr, GFP empty vector (EV), GFP-Peli1 WT, RING mutants, or phospho-dead mutant was transfected again into the cells. At 10 min post-microirradiation, fixed cells were immunostained with indicated antibodies. Scale bar, 10 μM. **b** Quantification of signals for GFP-Peli1 constructs, Peli1, and p-ATM at laser stripes. These plotted values represent mean±s.e.m. of 20 cells.

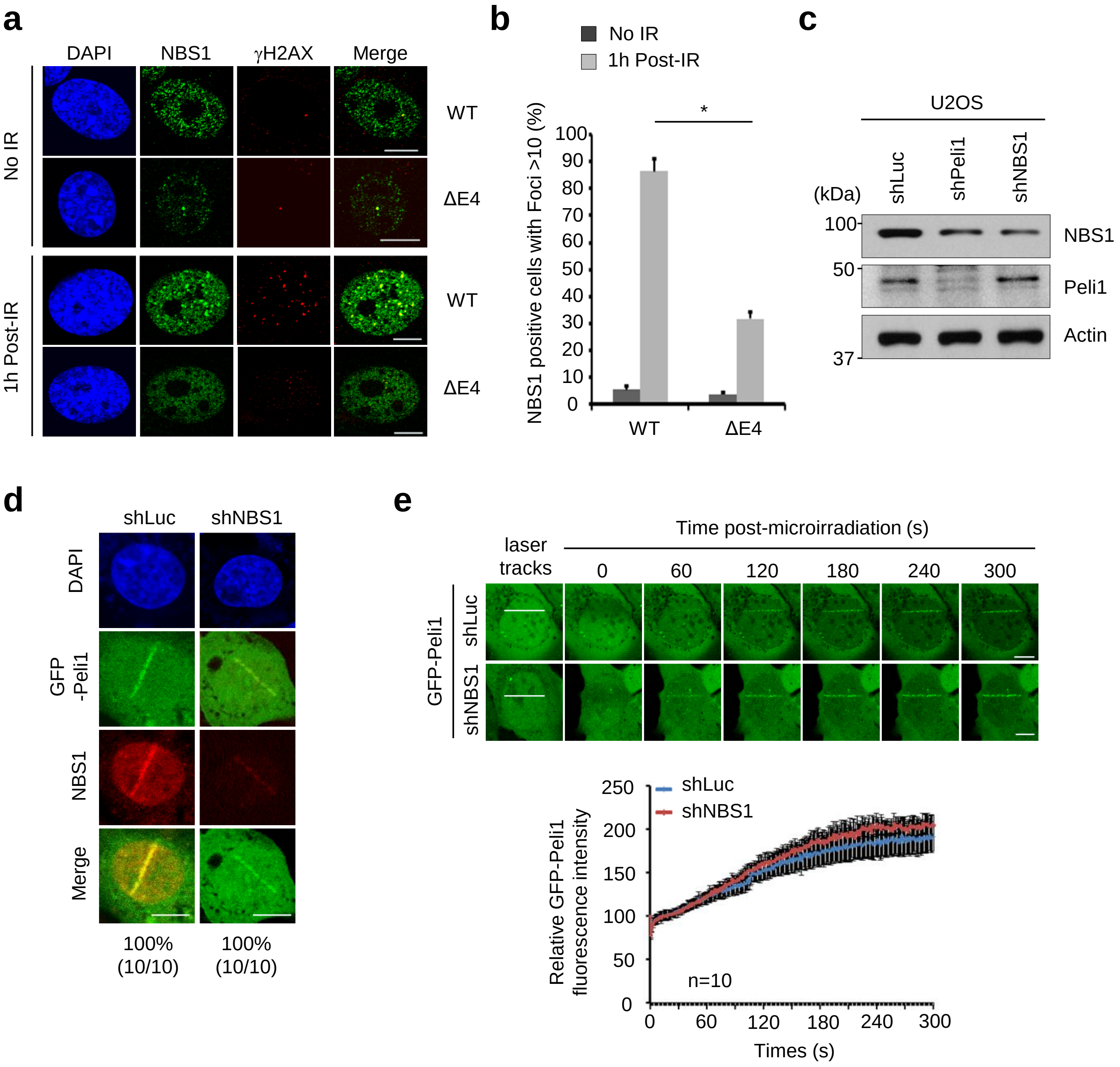

Supplementary Fig. 9 NBS1 is dispensable for Peli1 recruitment to DSB sites. **a, b** Peli1 WT and E4 truncated MEF cells were exposed to IR (4 Gy), fixed, and stained with anti-NBS1 and anti-γH2AX antibodies at 1 hr post-IR. Representative images of cells are shown in (**a**). Cells with multiple NBS1 foci (>10) were quantified indicated populations (**b**). Quantification results are presented as mean±s.e.m. of independent experiments. More than 100 cells were counted in each experiment. The scale bar represents 10 μM. Student's *t*-test was used for statistical analyses. **c** U2OS cells were transfected with shLuc, shPeli1 or shNBS1. At 48 hr post-transfection, cells were harvested and subjected to immunoblotting with anti-NBS1, anti-Peli1, and anti-actin antibodies. **d** U2OS cells were transfected with GFP-Peli1 together with shLuc or shNBS1. At 48 hr post-transfection, cells were subjected to laser microirradiation. Co-localization of Peli1 and NBS1 at laser-induced DNA lesions (10 min after laser microirradiation) is shown. Scale bar, 10 μM. **e** U2OS cells were transfected with GFP-Peli1 together with shLuc or shNBS1. At 48 hr post-transfection, cells were subjected to laser microirradiation. Laser stripes were examined at the indicated time point (upper panel). The intensity of each laser stripe in each time point was determined by averaging values from 10 cells and graphed (bottom panel). Scale bar, 10 μM

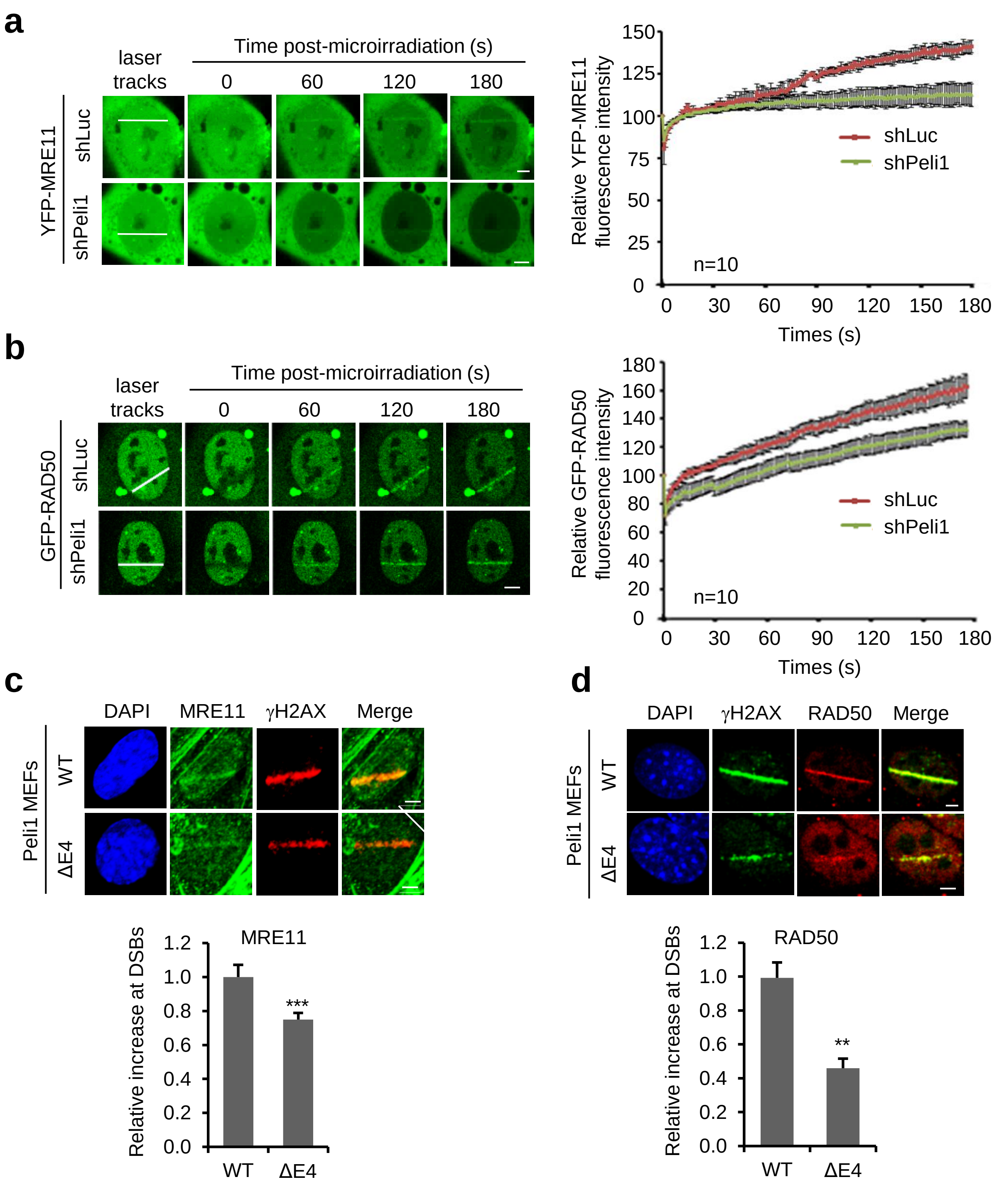

Supplementary Fig. 10 Peli1 is critical for MRE11 and RAD50 recruitment to DSB sites. **a, b** U2OS cells were transfected with YFP-MRE11 (**a**) or GFP-RAD50 (**b**) together with or without shPeli1. After 48 hr, cells were subjected to laser microirradiation. Laser stripes were examined at indicated time point. The intensity of each laser stripe in each time point was determined by averaging values from 10 cells and graphed in the right panel. Scale bar, 10 μM. **c, d** Peli1 WT and KO MEF cells were microirradiated with UV laser (10 min), fixed, and immunostained with anti-MRE11 (**c**), anti-RAD50 (**d**) antibodies. Staining of γH2AX was used as a positive control for accumulation at sites of laser tracks. Scale bar, 10 μM. Mean levels of indicated proteins accumulated at laser tracks were quantified using Image J software and plotted as indicated (each of lower panels). Data are presented as mean±s.e.m; n=20 cells. Student's *t*-test was used for statistical analyses.

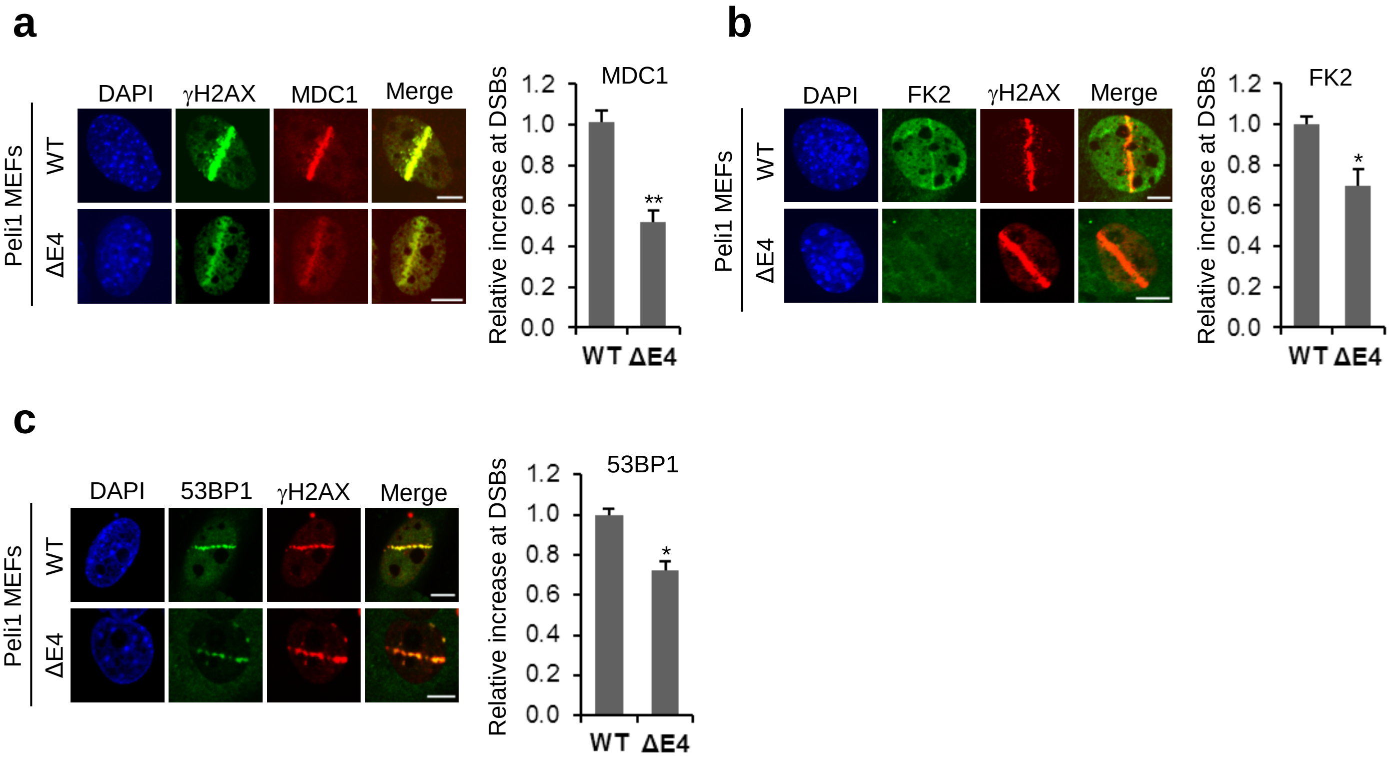

Supplementary Fig. 11 Recruitment of MDC1, FK2, and 53BP1 to DSB sites in Peli1-defective MEF cells. **a-c** Peli1 WT and KO MEF cells were microirradiated with UV laser (10 min), fixed, and immunostained with anti-MDC1 (**a**), anti-FK2 (**b**), and anti-53BP1 (**c**) antibodies. Staining of γH2AX was used as a positive control for accumulation at sites of laser tracks. The scale bar represents 10 μM. Mean levels of indicated proteins accumulated at laser tracks were quantified using Image J software and plotted as indicated (right panel). Data are shown as mean±s.e.m; n=20 cells. Student's *t*-test was used for statistical analyses.

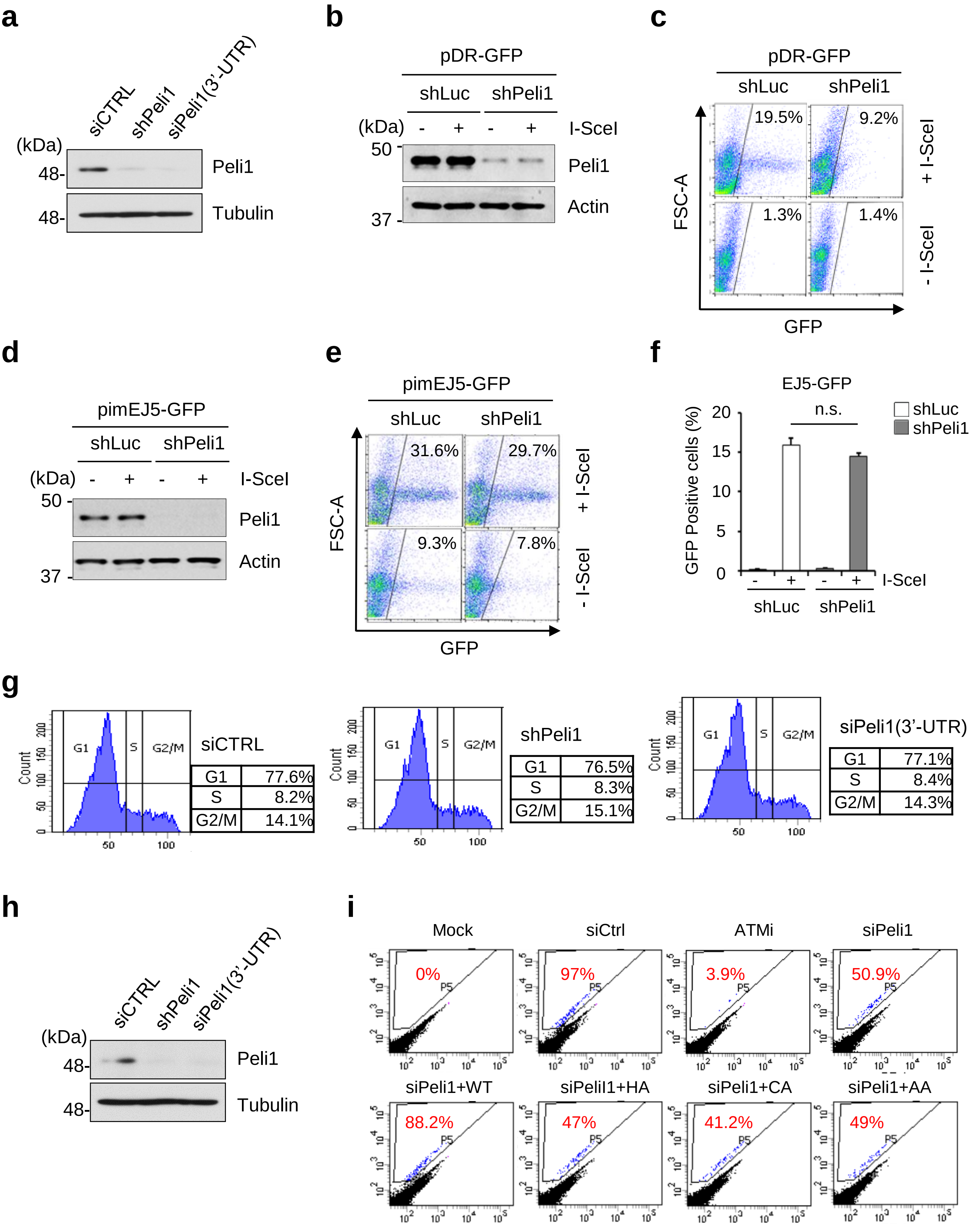
a
siCTRL
shPeli1
siPeli1(3'-UTR)
(kDa)
48-
Peli1
48-
Tubulin
b
pDR-GFP
shLuc
shPeli1
(kDa)
- + - +
I-SceI
50
Peli1
Actin
37
c
pDR-GFP
shLuc
shPeli1
19.5%
9.2%
1.3%
1.4%
+ I-SceI
- I-SceI
FSC-A
GFP
d
pimEJ5-GFP
shLuc
shPeli1
(kDa)
- + - +
I-SceI
50
Peli1
Actin
37
e
pimEJ5-GFP
shLuc
shPeli1
31.6%
29.7%
9.3%
7.8%
+ I-SceI
- I-SceI
FSC-A
GFP
f
EJ5-GFP
n.s.
shLuc
shPeli1
GFP Positive cells (%)
20
15
10
5
0
- + - +
I-SceI
shLuc
shPeli1
g
Count
G1
S
G2/M
50
100
siCTRL
G1 77.6%
S 8.2%
G2/M 14.1%
shPeli1
G1 76.5%
S 8.3%
G2/M 15.1%
siPeli1(3'-UTR)
G1 77.1%
S 8.4%
G2/M 14.3%
h
siCTRL
shPeli1
siPeli1(3'-UTR)
(kDa)
48-
Peli1
48-
Tubulin
i
Mock
siCtrl
ATMi
siPeli1
0%
97%
3.9%
50.9%
P5
siPeli1+WT
siPelil1+HA
siPeli1+CA
siPeli1+AA
88.2%
47%
41.2%
49%

Supplementary Fig. 12 Peli1 involves in HR repair, but not NHEJ. **a** Expression levels of Peli1 in DR-GFP stable cell lines. At 48 hr transfection of siCTRL, shPeli1, or siPeli1 3'-UTR-targeting siRNA, expression level of Peli1 was detected with anti-Peli1 antibody. **b, d** Expression levels of Peli1 with shPeli1 or shLuc in pDR-GFP (**b**) or pimEJ5-GFP (**d**) expressing 293T cells. **c, e** Twenty-four hours after transfection of either shLuc or shPeli1 vector in 293T cells expressing pDR-GFP or expressing pimEJ5-GFP, I-*Sce*I expression vector was transfected into these cells to generate a DSB within *Sce*-GFP. FACS analysis was carried out to quantify HR-repaired (**c**) or NHEJ-repaired (**e**) GFP^+ cells. **f** Twenty-four hours after transfection of either shLuc or shPeli1 vector in EJ5-GFP stable cell lines, the I-*Sce*I expression vector was transfected into these cells to generate a DSB within *Sce*-GFP. FACS analysis was carried out for HR-repaired GFP positive cells. n.s., not significant. Student's *t*-test was used for statistical analyses. **g, h** Cell cycle profile in depletion of Peli1 with shPeli1 or siPeli1 3'-UTR-targeting siRNA (**g**). Peli1 expression levels in depletion of Peli1 with shPeli1 or siPeli1 3'-UTR-targeting siRNA. Whole cell lysates were immunostained with indicated antibodies (**h**). **i** U2OS HR reporter stable cells were transfected with siRNA-targeting Peli1 3'-UTR region. After 24 hr, I-*Sce*I expressing vector as well as Myc-Peli1 WT, RING mutants, and phospho-dead mutant were transfected into reporter cells. After 48 hr, GFP-positive populations were analyzed by FACS. An ATM inhibitor (ATMi) was used as a HR inhibition positive control.

Supplementary Table 1. List of antibodies used in this study

Antibody	WB (Dilution)	IP (Dilution)	ICC (Dilution)	Catalog No.
γH2AX	1:5000	-	1:2000	Merk Millipore, 05-636
H2AX	1:1000	-	-	Cell signaling, 7631s
PELI1	1:1000	1:500	1:250	Abcam, ab199336
ACTIN	1:3000	-	-	Sigma-Aldrich, A2066
53BP1	-	-	1:250	Cell signaling, 4937s
GFP	1:1000	1:100	-	SantaCruz, sc-9996
TUBULIN	1:2000	-	-	SantaCruz, sc-23948
p-ATM (S1981)	1:500	-	1:250	Abcam, ab36810
ATM	1:1000	-	-	Calbiochem, 819-844
pSQ/TQ	1:500	-	-	Cell signaling, 6966s
HA	1:1000	-	-	SantaCruz, sc-7392
MRE11	1:1000	-	1:250	Abcam, ab214
CTIP	1:1000	-	-	SantaCruz, sc-271339
MDC1	1:1000	-	1:250	Merk Millipore, 05-1572
RFP	1:1000	-	-	Abcam, ab233
FLAG	1:5000	1:1000	1:1000	Sigma-Aldrich, F1804
RAD50	1:1000		1:250	GeneTex, GTX119731
NBS1	1:1000	1:500	1:250	Abcam, ab175800
MYC	1:1000	1:200	-	SantaCruz, sc-788
K63 Ub	1:500	-	-	Cell signaling, 5621s
UBC13	1:1000	-	-	SantaCruz, sc-376470
EXO1	1:1000	-	-	Cusabio, CSB-PA002455
p-BRCA1 (S1524)	1:1000	-	-	Cell signaling, 9009s
RAD51	1:1000	-	1:250	GeneTex, GTX100469
RPA32	1:1000	-	1:250	Abcam, ab2175
p-CHK1 (S317)	1:1000	-	-	Cell signaling, 12302s
p-CHK1 (S345)	1:1000	-	-	Cell signaling, 2348s
CHK1	1:1000	-	-	Cell signaling, 2360s
p-ATR (S428)	1:1000	-	-	Cell signaling, 2853s
ATR	1:1000	-	-	Cell signaling, 13934s
CPD	1:1000	-	-	Kamiya, MC-062
6-4PP	1:1000	-	-	Cosmo Bio, CAC-NM-DND-002
CYCLIN E	-	-	1:100	SantaCruz, sc-481
CYCLIN B1	-	-	1:100	SantaCruz, sc-245
FK2	-	-	1:200	Merk Millipore, 04-263
