# Supplementary material for: Pellino1 regulates reversible ATM activation via NBS1 ubiquitination at DNA double-strand breaks
Source: Nat Commun. 2019 Apr 5;10:1577. doi: 10.1038/s41467-019-09641-9 (PMC6450972; doi:10.1038/s41467-019-09641-9)

Uncropped raw immunoblots (related to Fig. 1, Fig. 2, Fig. 3, Fig. 4)

Fig. 1c

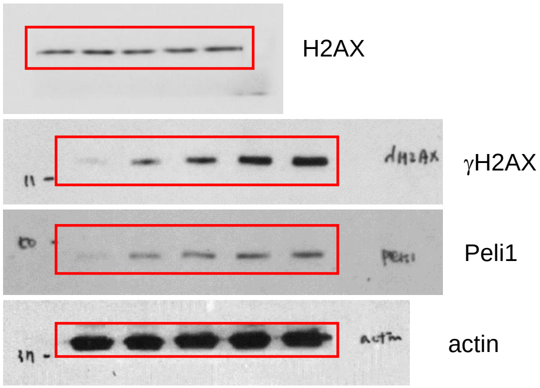

Fig. 2b

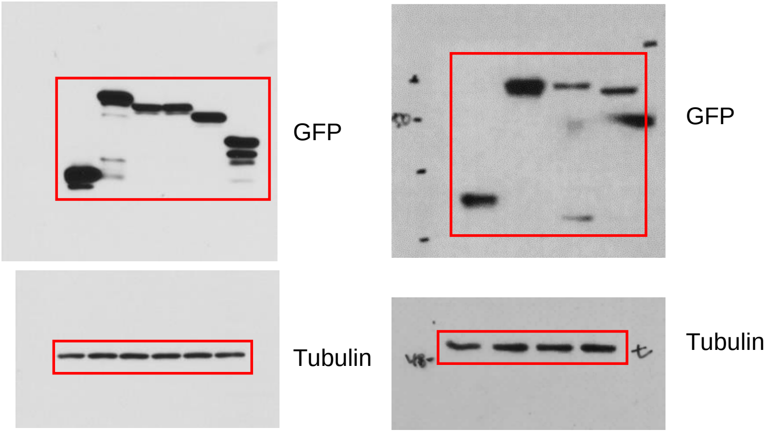

Fig. 2f

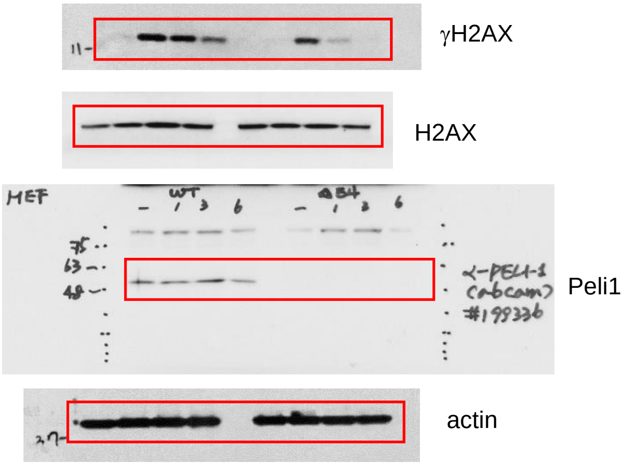

Fig. 3c

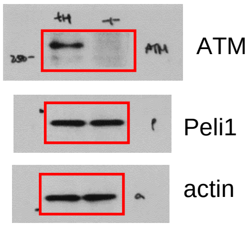

Fig. 3e

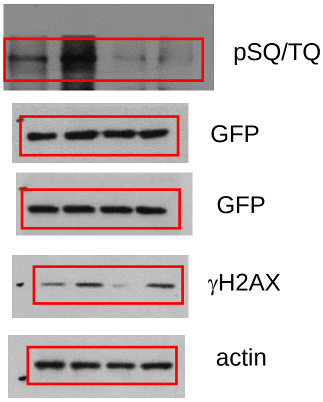

Fig. 3f

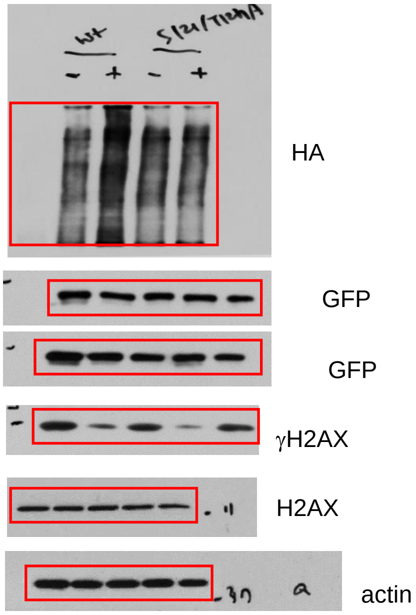

Fig. 3i

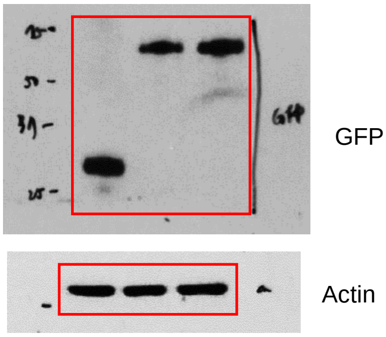

Fig. 4b

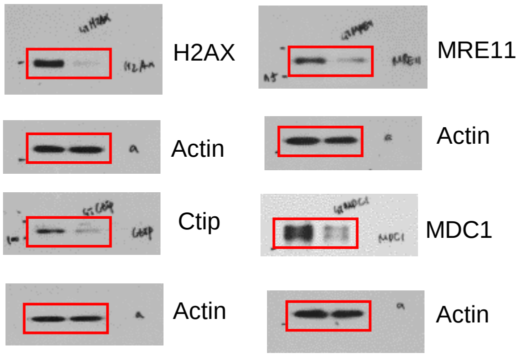

Fig. 4d

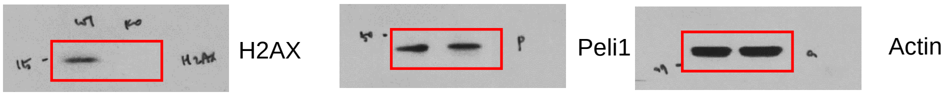

Fig. 4h

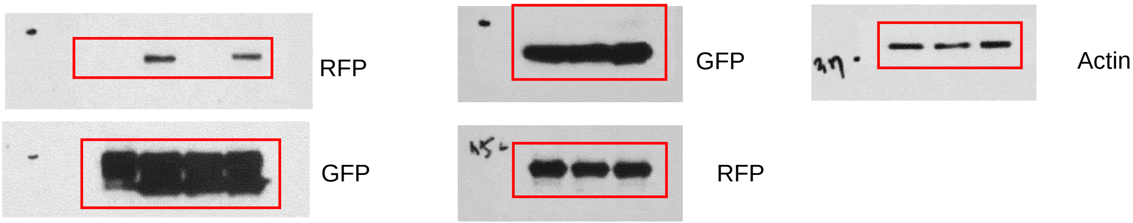

Uncropped raw immunoblots (related to Fig. 4, Fig. 5, Fig. 6)

Fig. 4i

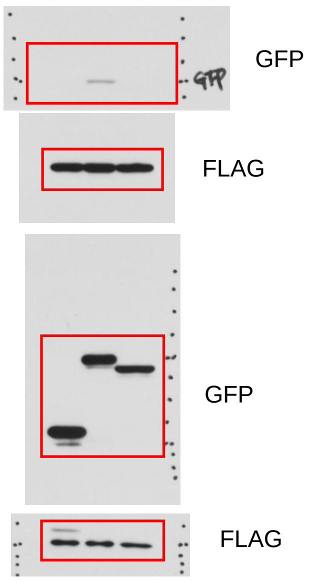

Fig. 5a

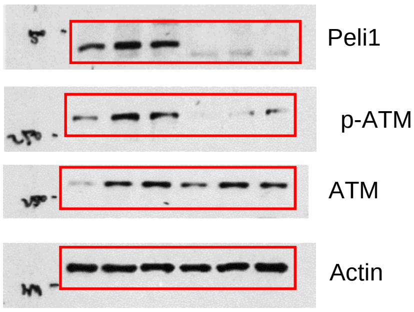

Fig. 5c

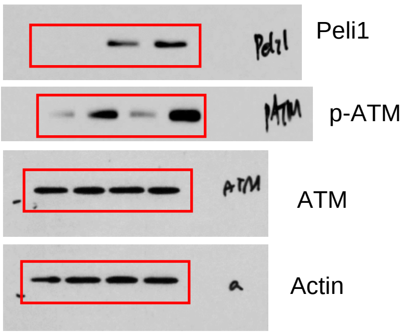

Fig. 5e

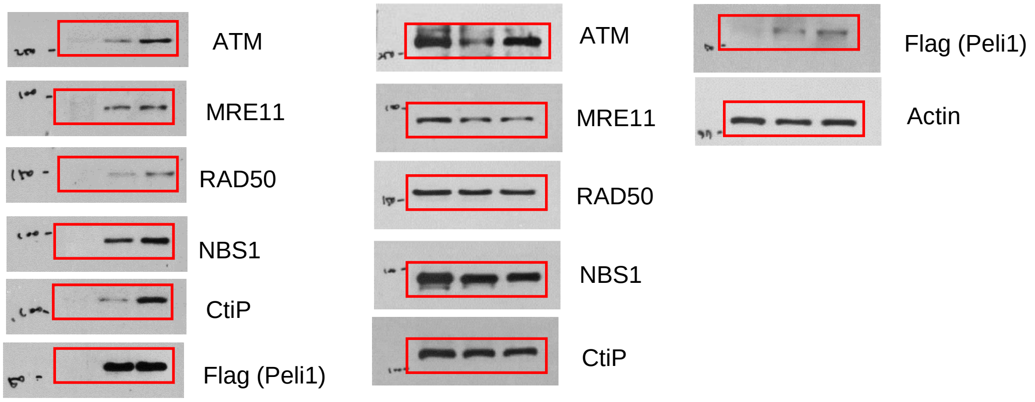

Fig. 5f

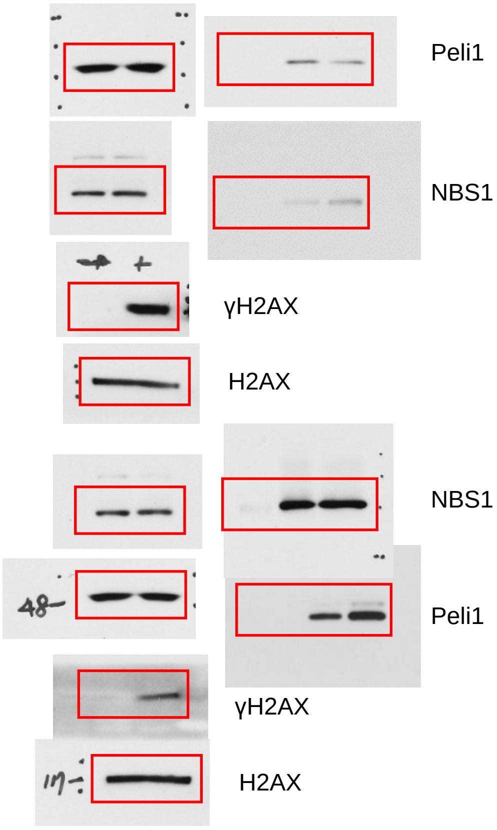

Fig. 6a

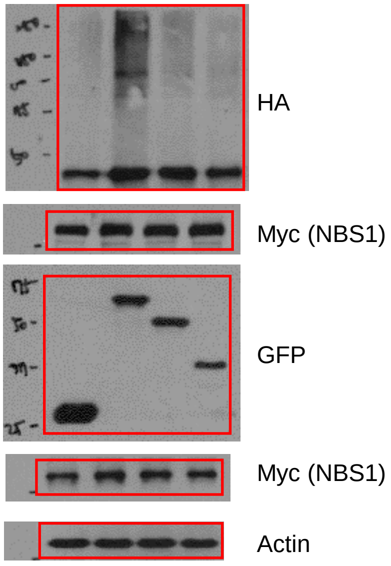

Fig. 6c

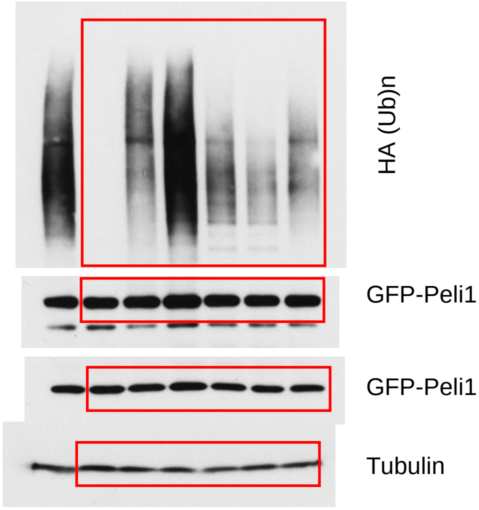

Fig. 6d

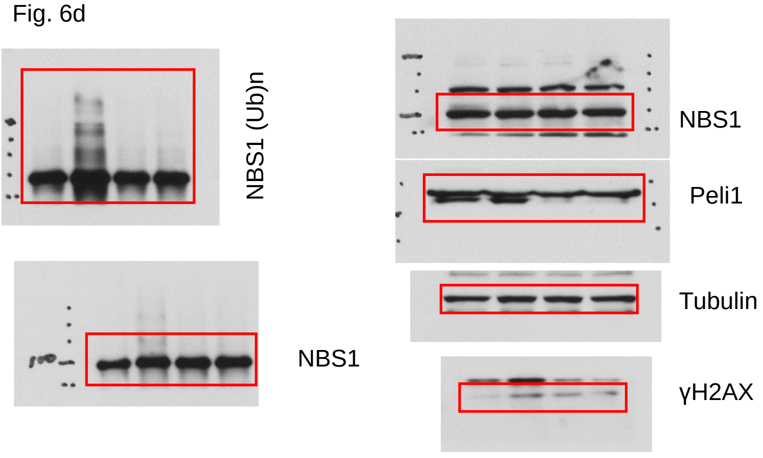

Uncropped raw immunoblots (related to Fig. 6, Fig. 7, Fig. 8)

Fig. 6e

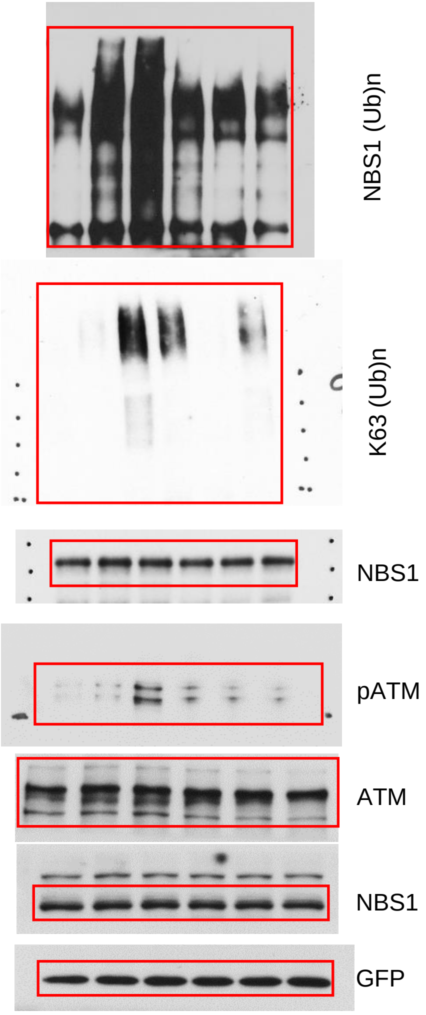

Fig. 6f

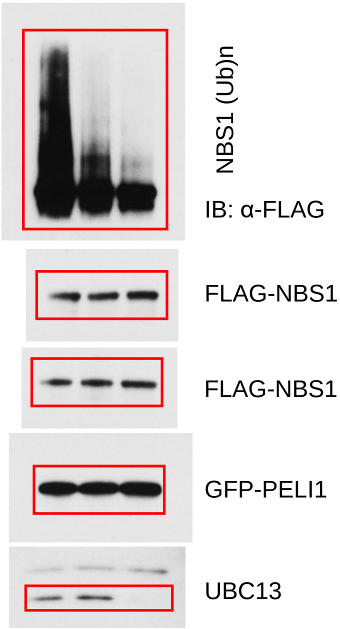

Fig. 7c

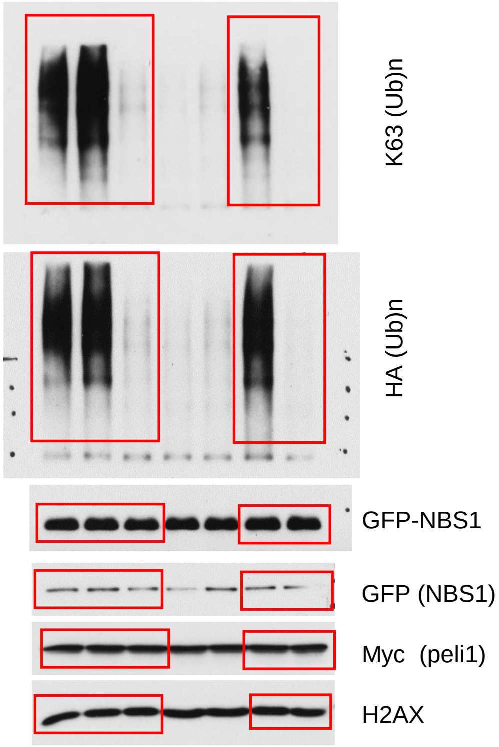

Fig. 7e

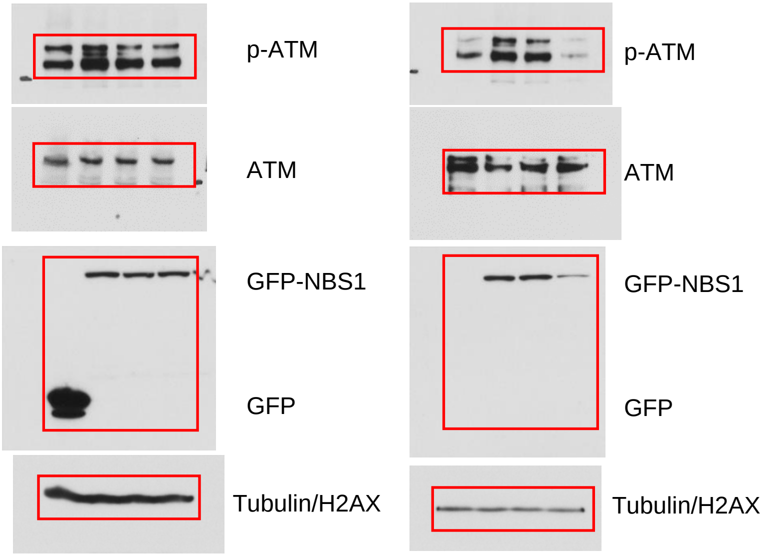

Fig. 8a

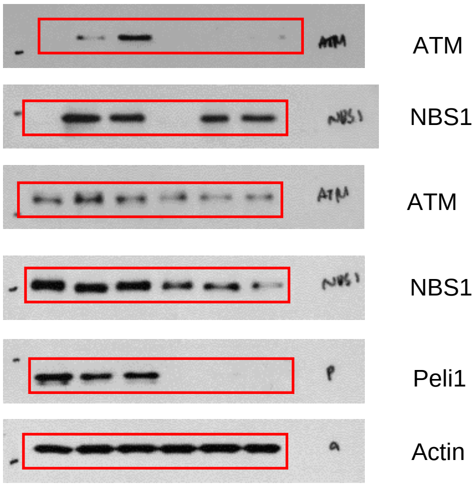

Fig. 8b

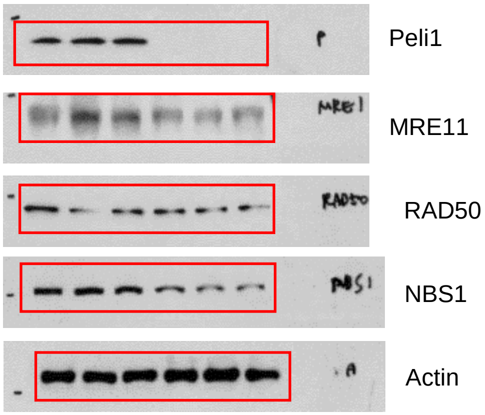

Fig. 8c

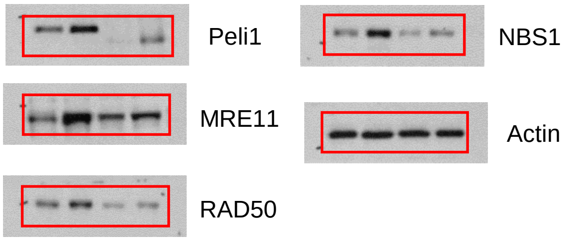

Uncropped raw immunoblots (related to Fig. 8, Fig. 9)

Fig. 8d

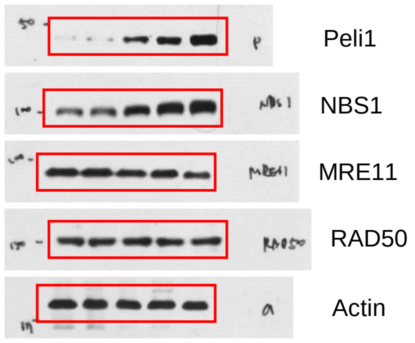

Fig. 9a

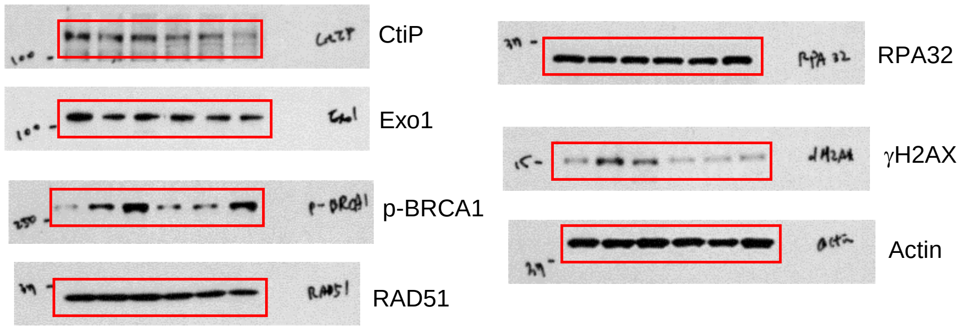

Uncropped raw immunoblots (related to Supplementary Fig. 1, Supplementary Fig. 2, Supplementary Fig. 6, Supplementary Fig. 7)

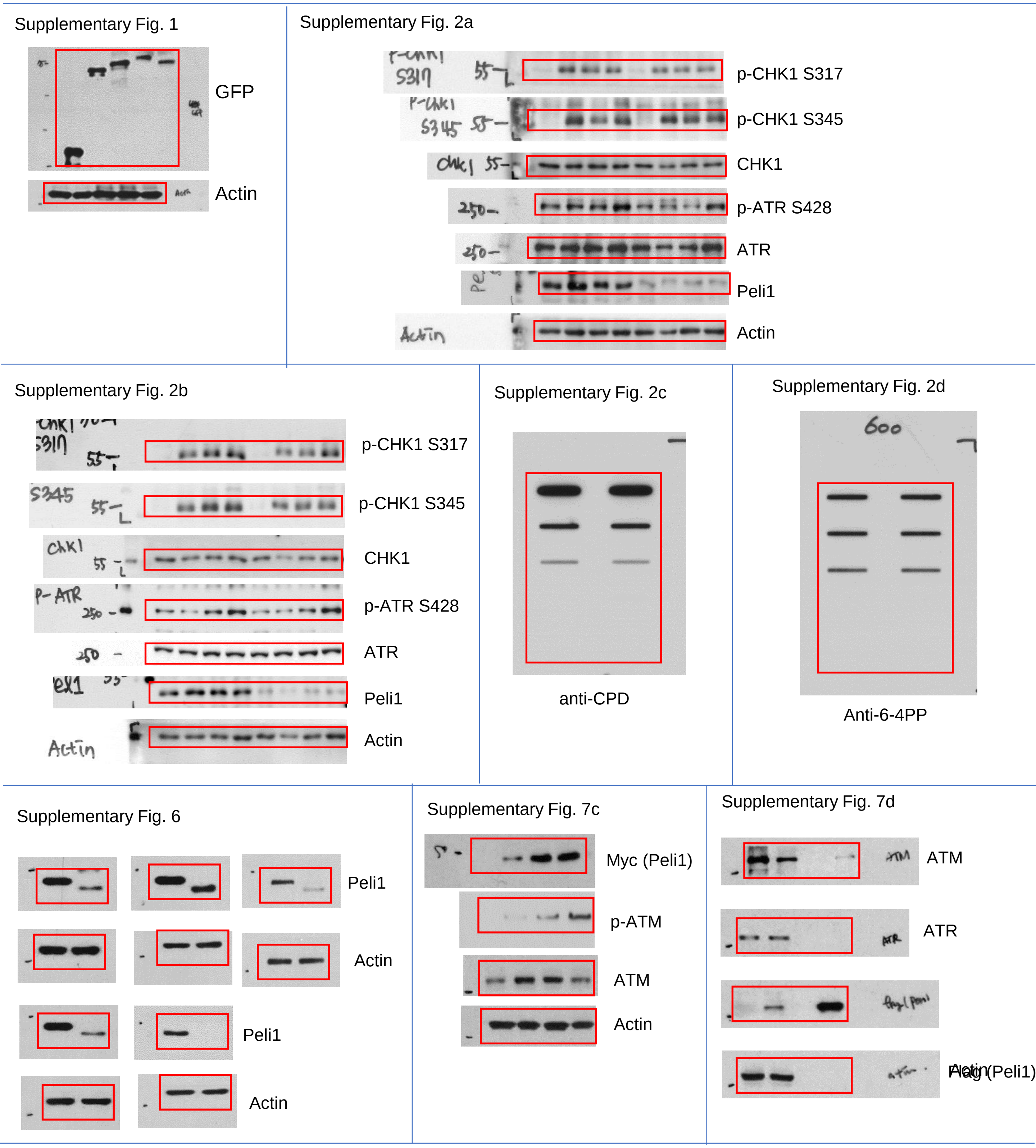

Uncropped raw immunoblots (related to Supplementary Fig. 8, Supplementary Fig. 10, Supplementary Fig. 13)

Supplementary Fig. 8b

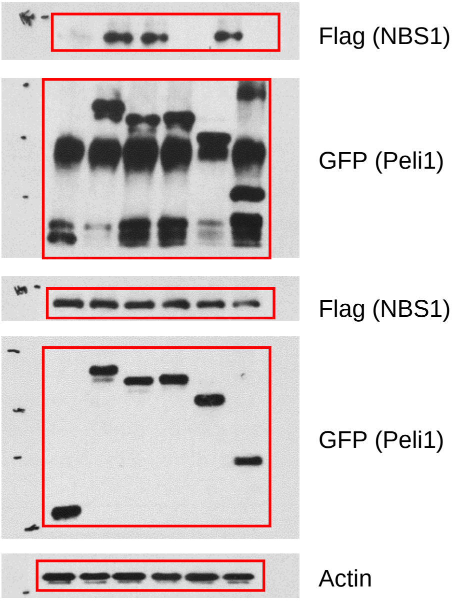

Supplementary Fig. 8d

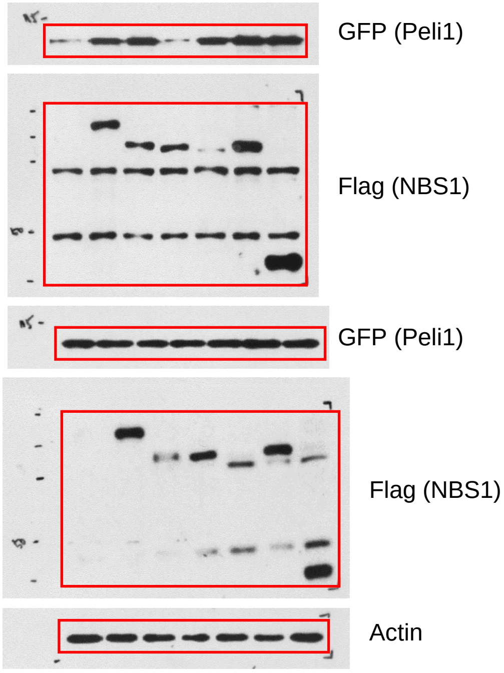

Supplementary Fig. 10c

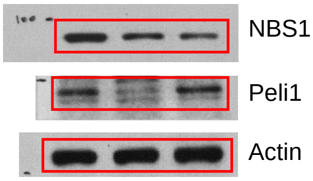

Supplementary Fig. 13a

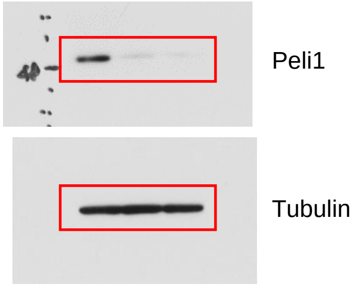

Supplementary Fig. 13b

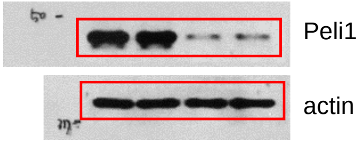

Supplementary Fig. 13d

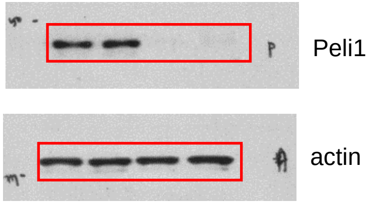

Supplementary Fig. 13h

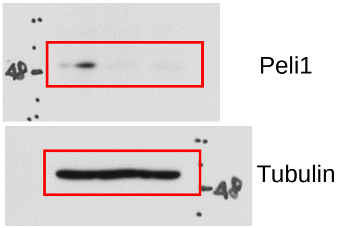

Supplement: Supplementary file 4 — Source Data [file 41467_2019_9641_MOESM4_ESM.zip › Source Data 2.pdf]
